# Grade 4 Neutropenia Secondary to Immune Checkpoint Inhibition — A Descriptive Observational Retrospective Multicenter Analysis

**DOI:** 10.3389/fonc.2021.765608

**Published:** 2021-10-21

**Authors:** Anne Zaremba, Rafaela Kramer, Viola De Temple, Stefanie Bertram, Martin Salzmann, Anja Gesierich, Lydia Reinhardt, Barouyr Baroudjian, Michael M. Sachse, Gunhild Mechtersheimer, Douglas B. Johnson, Alison M. Weppler, Lavinia Spain, Carmen Loquai, Milena Dudda, Claudia Pföhler, Adriana Hepner, Georgina V. Long, Alexander M. Menzies, Matteo S. Carlino, Céleste Lebbé, Tomohiro Enokida, Makoto Tahara, Paul J. Bröckelmann, Thomas Eigentler, Katharina C. Kähler, Ralf Gutzmer, Carola Berking, Selma Ugurel, Nadine Stadtler, Antje Sucker, Jürgen C. Becker, Elisabeth Livingstone, Friedegund Meier, Jessica C. Hassel, Dirk Schadendorf, Maher Hanoun, Lucie Heinzerling, Lisa Zimmer

**Affiliations:** ^1^ Department of Dermatology, Venerology and Allergology, University Hospital Essen, Essen, Germany; ^2^ Department of Dermatology, Universitätsklinikum Erlangen, Friedrich-Alexander-Universität Erlangen-Nürnberg (FAU), Erlangen, Germany; ^3^ Deutsches Zentrum Immuntherapie (DZI) , Erlangen, Germany; ^4^ Comprehensive Cancer Center Erlangen-European Metropolitan Area of Nürnberg (CCC ER-EMN), Erlangen, Germany; ^5^ Department of Dermatology, Mühlenkreiskliniken AöR, Ruhr University Bochum, Minden, Germany; ^6^ Institute of Pathology, University Hospital Essen, Essen, Germany; ^7^ Skin Cancer Center, Department of Dermatology and National Center for Tumor Diseases (NCT), University Hospital, Heidelberg, Germany; ^8^ Department of Dermatology, Venereology and Allergology, University Hospital Würzburg, Würzburg, Germany; ^9^ Skin Cancer Center at the University Cancer Centre Dresden and National Center for Tumor Diseases, Dresden, Germany; ^10^ Department of Dermatology, University Hospital Carl Gustav Carus, Technische Universität (TU) Dresden, Dresden, Germany; ^11^ Université de Paris, Department of Dermatology, AP-HP Hôpital Saint Louis, INSERM U976, Paris, France; ^12^ Department of Dermatology, Allergology and Phlebology, Bremerhaven Reinkenheide Hospital, Bremerhaven, Germany; ^13^ Institute of Pathology, University Hospital Heidelberg, Heidelberg, Germany; ^14^ Vanderbilt University Medical Center, Department of Medicine, Division of Hematology and Oncology, Nashville, TN, United States; ^15^ Medical Oncology Department, Peter MacCallum Cancer Centre, Melbourne, VIC, Australia; ^16^ Department of Dermatology, University Medical Center, Mainz, Germany; ^17^ Department of Dermatology, Saarland University Medical Center, Homburg, Germany; ^18^ Department of Medical Oncology, Melanoma Institute Australia, Sydney, NSW, Australia; ^19^ Medical Oncology Service, Instituto do Cancer do Estado de Sao Paulo, Sao Paulo, Brazil; ^20^ Melanoma Institute Australia, The University of Sydney, Sydney, NSW, Australia; ^21^ Faculty of Medicine and Health, The University of Sydney, Sydney, NSW, Australia; ^22^ Royal North Shore and Mater Hospitals, Sydney, NSW, Australia; ^23^ Westmead and Blacktown Hospitals, Sydney, NSW, Australia; ^24^ Department of Head and Neck Medical Oncology, National Cancer Center Hospital East, Chiba, Japan; ^25^ Department I of Internal Medicine, Centre of Integrated Oncology Aachen Bonn Cologne Duesseldorf (CIO ABCD), Faculty of Medicine and University Hospital of Cologne, University of Cologne, Cologne, Germany; ^26^ Department of Dermatology, University Hospital Tübingen, Germany and Charité – Universitätsmedizin Berlin, Corporate Member of Freie Universität Berlin and Humboldt Universität zu Berlin, Department of Dermatology, Venerology and Allergology, Berlin, Germany; ^27^ German Cancer Consortium (DKTK), Deutsches Krebsforschungszentrum (DKFZ), Heidelberg, Germany; ^28^ Department of Dermatology, University Hospital Schleswig Holstein, Kiel, Germany; ^29^ Department for Hematology and Stem Cell Transplantation, University Hospital Essen, Essen, Germany; ^30^ Department of Dermatology and Allergy, Ludwig-Maximilians-Universität (LMU), University Hospital, Munich, Germany

**Keywords:** malignant melanoma, immune checkpoint inhibition, adverse events, hematotoxicity, neutropenia

## Abstract

**Introduction:**

Immune checkpoint inhibitors (ICI) are increasingly being used to treat numerous cancer types. Together with improved recognition of toxicities, this has led to more frequent identification of rare immune-related adverse events (irAE), for which specific treatment strategies are needed. Neutropenia is a rare hematological irAE that has a potential for a high mortality rate because of its associated risk of sepsis. Prompt recognition and timely treatment of this life-threatening irAE are therefore critical to the outcome of patients with immune-related neutropenia.

**Methods:**

This multicenter international retrospective study was conducted at 17 melanoma centers to evaluate the clinical characteristics, diagnostics, treatment, and outcomes of melanoma patients with grade 4 neutropenia (<500 neutrophils/µl blood) treated with ICI between 2014 and 2020. Some of these patients received metamizole in addition to ICI (ICI+/met+). Bone marrow biopsies (BMB) of these patients were compared to BMB from non-ICI treated patients with metamizole-induced grade 4 neutropenia (ICI-/met+).

**Results:**

In total, 10 patients (median age at neutropenia onset: 66 years; seven men) with neutropenia were identified, equating to an incidence of 0.14%. Median onset of neutropenia was 6.4 weeks after starting ICI (range 1.4–49.1 weeks). Six patients showed inflammatory symptoms, including fever (n=3), erysipelas (n=1), pharyngeal abscess (n=1), and mucositis (n=1). Neutropenia was diagnosed in all patients by a differential blood count and additionally performed procedures including BMB (n=5). Nine of 10 patients received granulocyte colony-stimulating factors (G-CSF) to treat their grade 4 neutropenia. Four patients received systemic steroids (including two in combination with G-CSF, and one in combination with G-CSF and additional ciclosporin A). Four patients were treated with one or more antibiotic treatment lines, two with antimycotic treatment, and one with additional antiviral therapy. Five patients received metamizole concomitantly with ICI. One fatal outcome was reported. BMB indicated a numerically lower CD4+ to CD8+ T cells ratio in patients with irNeutropenia than in those with metamizole-induced neutropenia.

**Conclusion:**

Grade 4 neutropenia is a rare but potentially life-threatening side effect of ICI treatment. Most cases were sufficiently managed using G-CSF; however, adequate empiric antibiotic, antiviral, and antimycotic treatments should be administered if neutropenic infections are suspected. Immunosuppression using corticosteroids may be considered after other causes of neutropenia have been excluded.

## Introduction

Therapy with immune checkpoint inhibitors (ICI) that target the cytotoxic T-lymphocyte-associated protein-4 (CTLA-4; targeted by ipilimumab) and programmed cell death protein-1 (PD-1; targeted by pembrolizumab and nivolumab), either alone or in combination, achieves durable response rates in a variety of cancer types (1–3). Melanoma, a rare but aggressive skin cancer, is the lead indication for which ICI are approved (4–6). ICI have revolutionized the treatment of melanoma in metastatic settings and more recently in adjuvant and neoadjuvant settings (2, 7–9). As the indications for ICI have expanded, the number of patients treated in this way has risen. This trend is set to continue, because T-cell-targeted immunomodulators are currently used in combination with chemotherapies or as single agents in first- and second-line treatment of around 50 cancer types (10).

Because the use of ICI treatment is increasing, the diagnosis and management of not only common, but also rare, ICI-induced adverse events (AEs) is becoming more important. Adverse events affect 86–96% of patients and result from the loss of self-tolerance, leading to autoimmune-like events that can involve any organ (11). Hematological immune-related adverse events (irAEs) are estimated to affect less than 0.6-1% of ICI-treated patients (12, 13), but they are associated with a relatively high mortality rate of 2-14% (12–15). ICI-induced hematological side effects can affect all blood cell lineages. The most common hematological irAEs comprise anemia, thrombocytopenia, and neutropenia (12–14). In a recent publication, thrombocytopenia and leukopenia were the most common hematological irAEs, each affecting 34% of 50 patients who developed hematological irAEs induced by ICI, followed by neutropenia, which affected 28% of such patients (13).

Neutropenia describes a reduction of neutrophils to fewer than 1500 neutrophils per 1 µl of blood. Common Terminology Criteria for Adverse Events (CTCAE) grade 4 neutropenia, also termed agranulocytosis, involves a further, drug-related reduction of peripheral neutrophils and is defined by a reduction in the total number of neutrophils <500 per 1 µl of blood (16, 17). Sporadic acute grade 4 neutropenia is a very rare condition with an estimated incidence of 2–9 cases per million individuals per year (17). Previous studies have found that between 0% and 23% of cases of drug-induced grade 4 neutropenia are fatal (16). Patients may be completely asymptomatic; however, the classic clinical symptom triad associated with severe neutropenia consists of (i) angina tonsillitis, (ii) fever, and (iii) aphthous stomatitis (16). While neutropenia is a known and well-studied side-effect of intensive chemotherapy, the pathogenesis of conventional drug-induced neutropenia is not completely understood, although involvement of toxic or immunoallergic mechanisms is suspected (17). Apart from chemotherapy, the drugs most commonly associated with neutropenia are thiamazole, clozapine, sulfasalazine, and metamizole (17). Metamizole is a nonsteroidal anti-inflammatory drug (NSAID) with analgesic and antipyretic activity and belongs to the non-opioid analgesics (world health organization [WHO] stage 1). Although routinely used across Europe and Latin America, it has been identified as a disproportionately frequent trigger of grade 4 neutropenia (18). For this reason, it is effectively banned in the United States, Australia and several European countries (e.g. France, the United Kingdom) for pain management (18). Neutropenia has also been described as a fatal side effect of ICI (19). Because ICI-induced grade 4 neutropenia is so rare, only limited evidence exists regarding the incidence, diagnostics, and management of patients with this type of neutropenia. So far, one case series and one meta-analysis (n=34 patients) have studied immune-related neutropenia (irNeutropenia) (19, 20).

In this multicenter retrospective study, we sought to characterize ICI-induced grade 4 neutropenia in patients with advanced melanoma. Our evaluation included clinical signs and symptoms, diagnostic work up, hematopoietic development, treatments, and outcome. In addition, immunohistochemistry (IHC) was performed on bone marrow biopsies (BMB) from patients with ICI-induced grade 4 neutropenia and compared with IHC of BMB from patients with metamizole-induced grade 4 neutropenia, with particular focus on lymphocyte count.

## Patients and Methods

### Patients

We performed a descriptive observational multicenter retrospective study of melanoma patients who developed grade 4 neutropenia after exposure to ICI, which was recorded in a collected query of hematological side effects (13). Patients were screened between January 2014 and July 2020 at 17 tertiary referral centers in Europe, the United States, and Australia. To be included in the study, patients had to meet both of the following eligibility criteria: (1) diagnosis of grade 4 neutropenia according to the CTCAE (version 5.0) and (2) categorized as certainly or probably related to ICI therapy. Recovery was defined as restitution of neutrophil count. Patients were identified from the electronic medical records of the participating centers. To enable calculation of the incidence of grade 4 neutropenia, centers were asked to provide the total number of patients treated with ICI. Centers that could not state the total number of such patients were excluded from the incidence calculation. The following clinicopathological characteristics were collected for all patients: age, sex, melanoma mutational genotype, melanoma stage, systemic treatment, and specific data for neutropenia, i.e., differential blood count, neutrophil count, and BMB results and further diagnostics related to neutropenia. IHC was performed on BMB from patients with grade 4 ICI–metamizole (met)-induced neutropenia (ICI+/met+) and compared with IHC of BMB from non-ICI-treated patients with met-induced grade 4 neutropenia (ICI-/met+). Five non-ICI-treated patients with hematologically diagnosed met-induced grade 4 neutropenia (ICI-/met+) were identified from the electronic medical records of the Department of Hematology and Stem Cell Transplantation of the University Hospital Essen. Because the overall incidence of grade 4 neutropenia is low and prior ICI treatment had to be excluded, non-melanoma patients were selected as the comparison group for grade 4 met-induced neutropenia. Histological evaluation was performed by a local-board certified pathologist. The study was approved by the Ethics Committee of Duisburg-Essen University (19-9075-BO).

### Immunohistochemistry

IHC was performed by using the following antibodies:

**Table:** 

Antibody	Company	Purchase number	Host	Clonality	Clone	Secondary antibody	Dilution
CD3	CDS	CI597C01	Rabbit	Monoclonal	SP7	OptiView DAB IHC Detection Kit (Roche Diagnostics; IN, USA)	1:200
CD8	Dako	M7103	Mouse	Monoclonal	C8/144B	OptiView DAB IHC Detection Kit	1:150
CD4	Zytomed	503-3352	Rabbit	Monoclonal	SP35	OptiView DAB IHC Detection Kit	1:50
CD20	Roche	5267099001	Mouse	Monoclonal	L26	OptiView DAB IHC Detection Kit	Ready to use

Clusters of differentiation (CD) 20, CD3, CD4, and CD8 were stained using the Ventana Benchmark Ultra platform (Roche Diagnostics). Hematoxylin and eosin staining, Giemsa staining, and naphthol AS-D chloroacetate esterase (ASDCL) were used in accordance with institutional standards. For each sample, the number of positive cells in an area measuring 6.25 mm^2^ (10 high-power fields, defined by a field of view of 400× magnification) was counted manually by two blinded independent physicians of the Institute of Dermatology and Dermatohistopathology, University Hospital Essen. The total number of cells in the ICI–met-induced (ICI+/met+) and non-ICI met-induced (ICI-/met+) grade 4 neutropenia BMB samples were compared using the Mann–Whitney U test. One patient treated with ICI and metamizole (ICI+/met+) and concomitant B-cell chronic lymphocyte leukemia was excluded from CD20+ cell calculation.

## Results

### Clinical Characteristics of Patients With irNeutropenia (ICI+/met+ and ICI+/met-)

Over a period of 6.5 years between 2014 and 2020, more than 6961 melanoma patients were treated with ICI in 17 cancer centers. Two of these 17 centers were unable to specify the total number of ICI-treated patients. Ten patients (seven men, three women) experienced grade 4 neutropenia (**Table 1**). The incidence was 0.14%, with two centers excluded from the calculation of incidence. Median age at onset of grade 4 irNeutropenia was 66 years (range 28–80). Eight melanoma patients received neutropenia-triggering ICI for advanced disease (ipilimumab plus nivolumab n=7; pembrolizumab n=1), and two patients as adjuvant treatment (pembrolizumab n=1; nivolumab n=1). Five patients had received prior systemic therapy (adjuvant interferon α n=1; PD-1 monotherapy followed by chemotherapy n=1 [stopped 4 months before irNeutropenia]; PD-1 monotherapy within a clinical trial n=1; BRAF and MEK inhibition n=2, **Table 1**). Four patients had hematological comorbidities at the start of ICI therapy, comprising one patient each with the following: B-cell chronic lymphocytic leukemia (B-CLL), monoclonal gammopathy of unknown significance (MGUS), systemic mantle cell lymphoma, and pre-existing non-ir thrombocytopenia and lymphopenia. None of these patients received therapy for the respective hematological comorbidities when starting ICI nor suffered from neutropenia.

**Table 1:** Clinical characteristics of patients with irNeutropenia.

	Patient 1	Patient 2	Patient 3	Patient 4	Patient 5	Patient 6	Patient 7	Patient 8	Patient 9	Patient 10
*Age at neutropenia onset, years/sex*	28.4/F	53.4/M	60.2/M	69.9/M	71.7/M	79.8/M	70.7/F	62.2/M	74.1/M	38.7/F
*Checkpoint* *inhibitor(s) inducing neutropenia* *and dosage(s)*	Ipi (3 mg/kg) + nivo (1 mg/kg)	Pembro (2 mg/kg BW)	Ipi (3 mg/kg) + nivo (1 mg/kg)	Ipi (3 mg/kg) + nivo (1 mg/kg)	Ipi (3 mg/kg) + nivo (1 mg/kg)	Ipi (3 mg/kg) + nivo (1 mg/kg)	Ipi (3 mg/kg) + nivo (1 mg/kg)	Nivo (3 mg/kg BW)	Ipi (3 mg/kg) + nivo (1 mg/kg)	Pembro (200 mg/kg)
*Best response* *to therapy*	PD	PR	PD	CR	PD	PR	PD	PD (DD lymphoma)	PD	No PD
*Hematological comorbidities*	–	–	–	B-CLL	Thrombocytopenia (not immune-related); lymphopenia	MGUS	–	Systemic mantle cell lymphoma	–	–
*Melanoma type*	Skin	Skin	Mucosal	Skin	Skin	Skin	Mucosal	Skin	Skin	Skin
*Mutation status*	*BRAF* wt	*NRAS* mut	*BRAF* wt	*BRAF* wt	*BRAF* V600R	*NRAS* mut	*BRAF* wt	*BRAF* K601N	*BRAF* V600E	*BRAF* wt
*Adjuvant therapy*	Yes (IFN)	–	–	–	–	–	–	Yes (Nivo monotherapy)^#^	–	–
*Systemic therapies prior to ICI inducing neutropenia*	–	–	PD-1 monotherapy chemotherapy	–	BRAF+MEK inhibitor	–	Nivolumab monotherapy*	–	–	BRAF+MEK inhibitor**
*Melanoma stage at start of ICI inducing neutropenia*	IV	IV	IV	IV	IV	IV	IV	IIIC	IV	IIIB
*LDH elevated at ICI start*	Yes	No	No	No	Yes	No	No	No	No	No
*Other irAEs*	Thyroiditis Hepatitis	Vitiligo Arthritis Nephritis	Pancytopenia	Exanthema Diarrhea	Fatigue Fever	DM type III Hypophysitis Endogenous endophthalmitis Thrombocytopenia Neutropenia	None	Exanthema	Colitis	None
*Survival status*	Unknown^##^	Dead	Dead***	Alive	Dead	Dead	Alive	Alive	Dead	Alive

BW, bodyweight; CLL, chronic lymphocytic leukemia; CR, complete response; DD, differential diagnosis; DM, diabetes mellitus; F, female; ICI, immune checkpoint inhibitors; IFN, interferon α; ipi, ipilimumab; ir-, immune-related; irAE, immune-related adverse event; LDH, lactate dehydrogenase; M, male; MGUS, monoclonal gammopathy of unknown significance; mut, mutation; nivo, nivolumab; pembro; pembrolizumab; PD, progressive disease; PD-1, programmed death ligand 1; PR, partial response; wt, wildtype.

*within a clinical trial.

** not for melanoma, for ganglioglioma.

***due to neutropenia.

^#^inducing neutropenia.

^##^alive at last contact, lost to follow-up (returned to home country).

### Characteristics of Patients With Met-Induced Neutropenia (ICI-/met+)

The ICI-/met+ patients in the comparator cohort were all female (n=5) with a median age of 64 years (range 22–89) at diagnosis of grade 4 neutropenia (**Supplemental Tables 1**, **2**). All patients had BMB for diagnostic purposes.

### Clinical Course and Diagnostics of Grade 4 irNeutropenia

**Figure 1:**
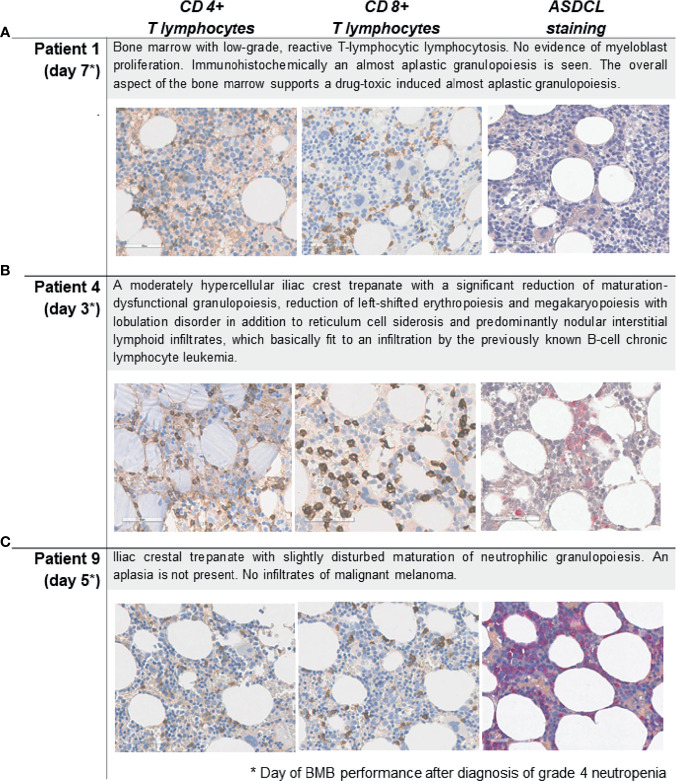
Bone marrow staining for CD4+ T and CD8+ T lymphocytes, and naphthol AS-D chloroacetate esterase (ASDCL) staining for myeloid cells in patients with ICI-induced grade 4 irNeutropenia (n=3). **(A)** Patient 1, **(B)** Patient 4, **(C)** Patient 9. Original magnification 400×. BMB, bone marrow biopsy; CD, cluster of differentiation.

The median time from starting ICI to onset of grade 4 neutropenia was 6.4 weeks (range 1.4–49.1). Two patients showed a decrease in cell number in more than one hematological lineage (Pat 3, Pat 6). Six patients received comedication at the time of grade 4 neutropenia, of whom five received metamizole. Additional medications started within 6 weeks before neutropenia onset are shown in **Table 2**. Six of the 10 patients showed inflammatory symptoms including fever (n=3), erysipelas (n=1), pharyngeal abscess formation (n=1), and mucositis (n=1). Two patients presented with unspecific symptoms (loss of appetite n=1; weakness and abdominal pain n=1). Eight patients had additional irAEs. These included colitis/diarrhea (n=2), endocrine AEs (n=2), exanthema (n=2), and hepatitis (n=1). Concurrent AEs occurred before (median 3.9 weeks before irNeutropenia onset n=7, [range 0.4–11.1]) and after (median 2.3 weeks, n=1) irNeutropenia first occurred. All patients developed grade 4 neutropenia with <500 neutrophils per µl of blood. Neutropenia was diagnosed in all patients by a differential blood count (**Table 3**). In individual cases a myelogram (n=1) or autoantibody test and cytogenetic analysis (n=1) was also performed. A BMB was performed in five patients, of whom three (Patients 1, 4, 9) were treated with concomitant metamizole (ICI+/met+; **Table 2** and **Figure 1**). All BMB showed abnormalities in neutrophilic granulopoiesis. No patient showed melanoma infiltration of the bone marrow. Immunohistochemistry of BMB from patients with ICI–met-induced grade 4 neutropenia (ICI+/met+) showed a range of immunohistochemical features (**Figure 1**), including impaired maturation of neutrophilic granulopoiesis (Pat 9) and depletion of granulocytes (Pat 4). The ratio of CD20+ to CD3+ T cells was similar between the ICI+/met+ and ICI-/met+ patients (0.24 vs. 0.23 CD20+ T cells per CD3+ T cell), while the ratio of CD4+ to CD8+ T cells was numerically lower in patients with ICI–met-induced neutropenia (ICI+/met+) than in patients with non-ICI met-induced neutropenia (ICI-/met+) (median 0.68 *vs*. 0.8 CD4+ T cells per CD8+ T cell, **Supplemental Figure 1**).

**Table 2:** Diagnostic methods and treatment of patients with irNeutropenia.

	Patient 1	Patient 2	Patient 3	Patient 4	Patient 5	Patient 6	Patient 7	Patient 8	Patient 9	Patient 10
*Time to neutropenia* *onset after ICI start, weeks*	7.3	49.1	6.6	4.6	6.9	18.6	6.1	1.6	2.9	1.4
*Additional drugs (started within 6 weeks prior onset of neutropenia)*	**Metamizole** Denosumab Dimenhydrinate Propranolol Prednisolone	Clexane Cotrimoxazole Pantoprazole Piperacillin/ tazobactam Methyl-prednisolone Hemodialysis	None	**Metamizole**	None	Hydrocortisone	**Metamizole**	None	**Metamizole**	**Metamizole** Lamotrigine Opipramol Ibuprofen Pantoprazole
*Signs and* *symptoms of neutropenia*	Weakness, Erysipelas, Pain	Fever, Weakness	Fever	Mucositis	Fever	Loss of appetite	Pharyngeal abscess	None	None	Weakness, Abdominal pain
*Diagnosis* *of neutropenia*	Lab BMB	Lab BMB	Lab Myelogram	Lab BMB	Lab	Lab BMB	Lab ENT examination	Lab	Lab Autoantibodies BMB Cytogenetic analysis	Lab
*Systemic steroids*	Prednisolone 1 mg/kg/d (80 mg) IV	Prednisolone 80 mg IV	None	None	Dexamethasone 8 mg IV 3×/d; Methylprednisolone 1 mg/kg IV, Methylprednisolone 1 mg/kg p.o.	Prednisolone 20 mg p.o.*	None	None	None	None
*Additional treatment*	Ciclosporin (175 mg/d) Piperacillin/tazobactam Vancomycin Ciprofloxacin G-CSF (Filgrastim 30 million IU/day SC) Valaciclovir (1000 mg/d) Fluconazole (100 mg/d)	Amphotericin B G-CSF (Filgrastim 30 million IU/d for 3 days)	G-CSF (Neupogen)	G-CSF	G-CSF (Granocyte SC 1×/d) Piperacillin/tazobactam (4×4.5 g IV) Levofloxacin (500 mg 1×/d)		G-CSF (Neupogen 30 million IU 1× daily for 6 d) Ciprofloxacin (500 mg 1-0-1 for 10 days) Unacid 3 g 1-1-1 (7 d) Amphotericin B	G-CSF	G-CSF Ciprofloxacin	G-CSF
*Outcome of neutropenia*	Resolved	Resolved	Patient died	Resolved	Resolved	Resolved	Resolved	Resolved	Resolved	Resolved
*Re-exposure with irAE-inducing ICI*	No	Yes (Ipilimumab)	No	No	No	No	Yes (Nivolumab)	Yes (Nivolumab)	No	Yes (Pembrolizumab)
*irAE after re-exposure*	–	Arthritis Vitiligo	–	–	–	–	None	Exanthema	–	None

BMB, bone marrow biopsy; ENT, ear-nose-throat; G-CSF, granulocyte colony-stimulating factors; ICI, immune checkpoint inhibition; ipi, ipilimumab; ir-, immune-related; irAE, immune-related adverse event; IV, intravenously; lab, laboratory diagnostics; nivo, nivolumab; SC, subcutaneously; pembro, pembrolizumab.

*started before grade 4 neutropenia.

**Table 3:** Blood counts before neutropenia onset and during maximum irNeutropenia.

	Patient 1	Patient 2	Patient 3	Patient 4	Patient 5	Patient 6	Patient 7	Patient 8	Patient 9	Patient 10
***Counts before neutropenia onset***										
*Hb in g/dl*	9.7	15.6	11.1	16.1	14.3	7	13.3	12.8	12.5	8.8
*Thrombocytes/nl*	533	209	427	156	134	280	230	182	127	485
*Leukos/nl*	10.76	4.7	6.36	7.8	3.69	7	4.89	3.1	5.83	6.6
*Neutros/nl*	8.64	2.9	4.87	3.74	2.45	3.3	3	2.1	4.26	5
***Counts during maximum neutropenia***										
*Hb in g/dl*	9.4	11.5	7.7	12.8	12.7	7.9	11.5	11.8	10.6	8.5
*Thrombocytes/nl*	366	166	137	329	148	0.3	312	192	175	344
*Leukos/nl*	0.3	0.98	1.39	2.2	0.92	4.3	0.99	2.36	1.76	1.5
*Neutros/nl*	Nm**	Nm**	0.02	0.01	0.05	0.3	0.13	0.4	0*	0.06
***% Neutrophil decrease*** ***from baseline***	>99	>99	99.59	99.73	97.96	90.91	95.67	80.95	>99	98.80

hb, hemoglobin; ir-, immune-related; leukos, leukocytes; neutros, neutrocytes; nm, not measurable.

*Segmented-cored.

**Insufficient cells to detect and count types of leukocytes in differential blood count.

### Management and Outcome of Grade 4 ICI-Induced Neutropenia

**Figure 2:**
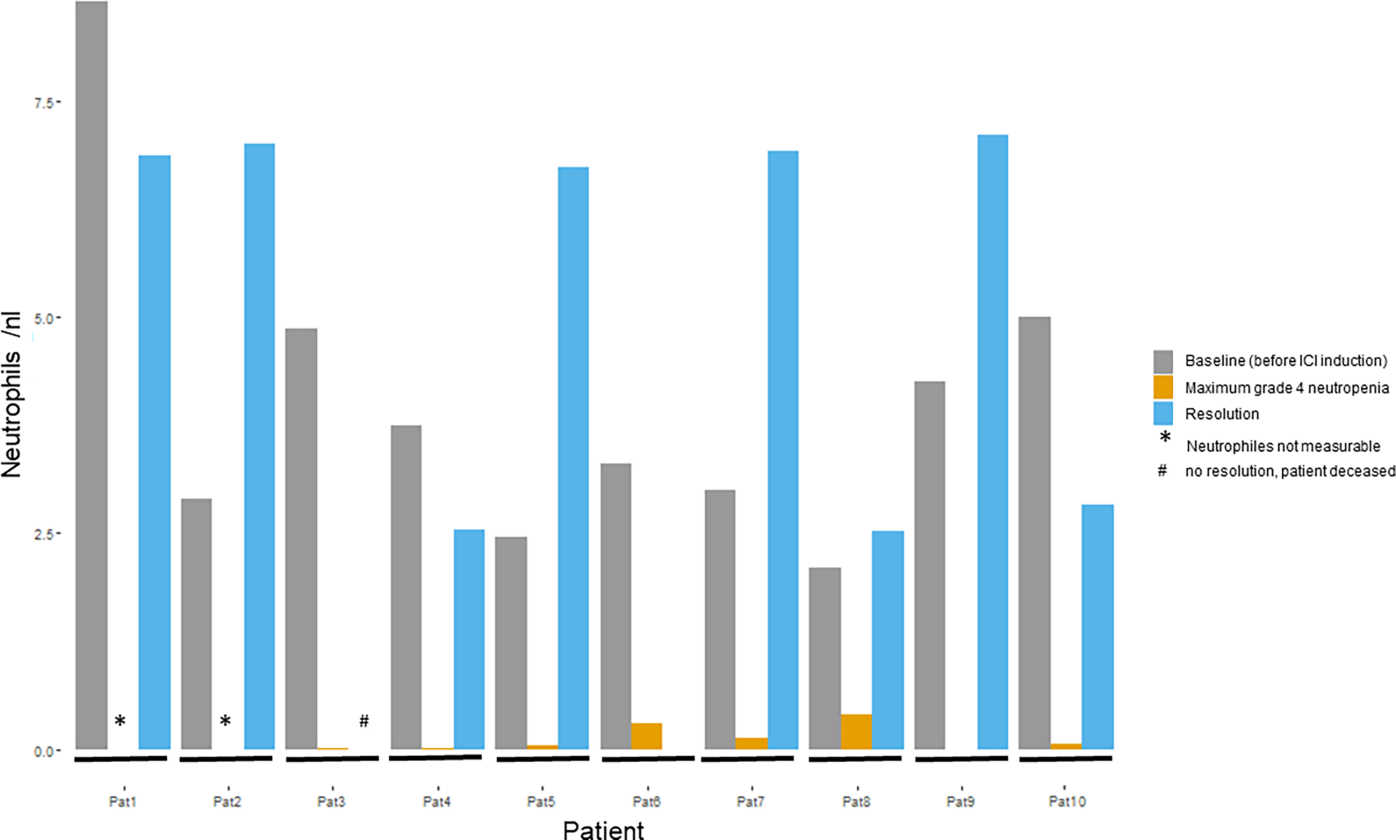
Course of neutrophil granulocytes in patients with grade 4 irNeutropenia (n=10). Figure shows neutrophil values in nl at baseline (before ICI induction), for maximum grade 4 neutropenia, and, if it occurred, after resolution of neutropenia. ICI, immune checkpoint inhibitors; nl, nanoliter.

**Figure 3:**
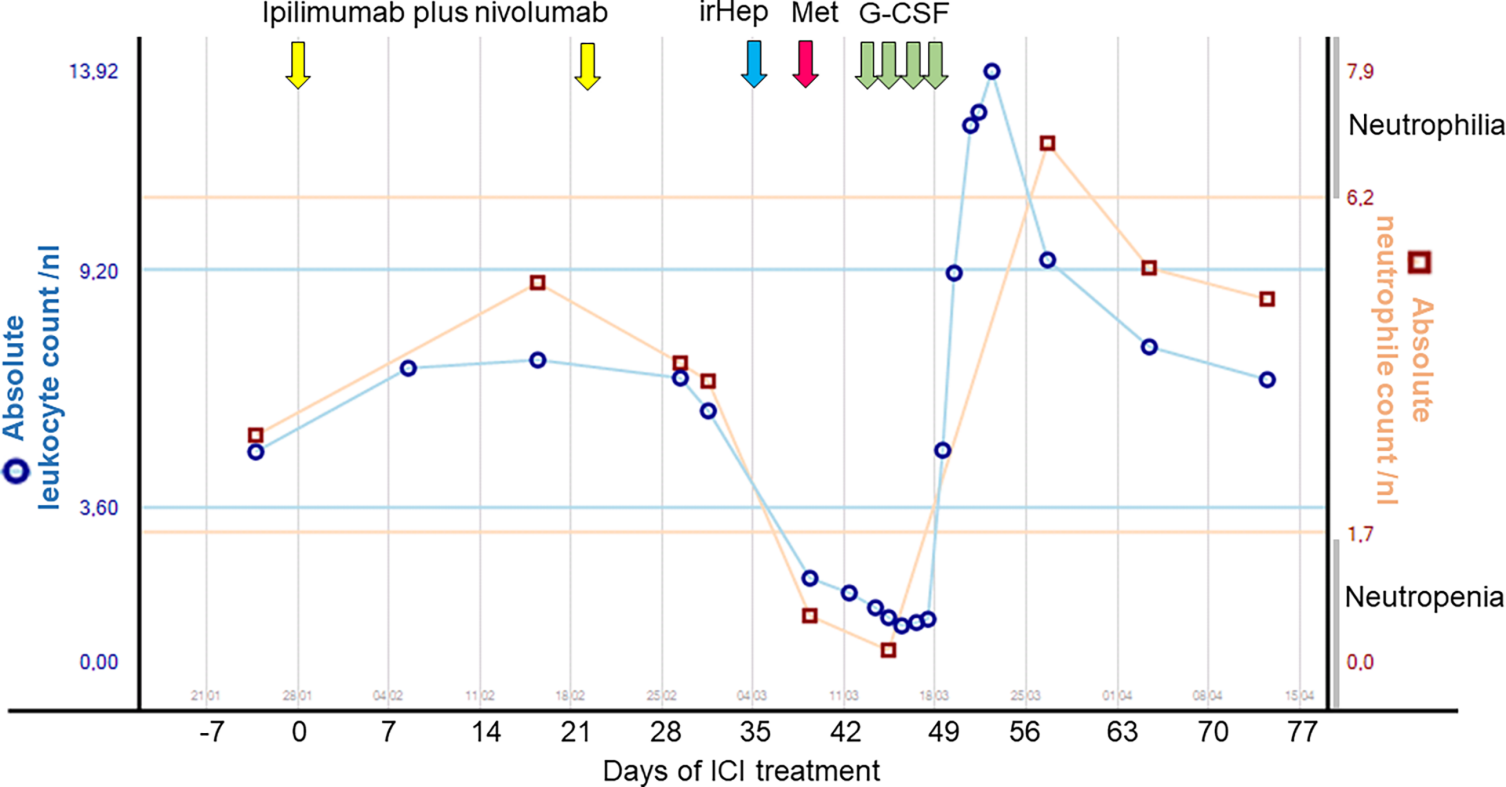
Timeline of patient 7: Neutrophil and leukocyte counts over time following administration of combined ipilimumab–nivolumab and metamizole as well as subsequent treatment interventions. G-CSF, granulocyte colony-stimulating factors; Hep-, hepatitis; ICI, immune checkpoint inhibitor therapy; ir-, immune-related; Met, metamizole; nl, nanoliter.

The median duration of grade 4 neutropenia among the 10 ICI-treated patients was 9.5 days (range 3–32; **Supplemental Table 1**) and seven patients were hospitalized for treatment. In four patients, neutropenia was complicated by infection. Nine patients showed a maximum neutrophil reduction of >90% from baseline (**Table 3** and **Figure 2**). One patient (Pat 6) had simultaneous thrombocytopenia and worsening of pre-existing anemia, one patient showed a pancytopenia (Pat 3) (**Table 3**). Nine patients received granulocyte (macrophage) colony-stimulating factors (G-CSF) subcutaneously to treat their grade 4 neutropenia. Four patients received systemic steroids (three of whom received concomitant G-CSF). The dosage of systemic steroids varied: One patient received 50 mg prednisolone equivalent three times daily, one patient 20 mg prednisolone once daily, and two patients received bodyweight-adapted 2.5 mg prednisolone per kilogram once daily as initial dose. One patient (Pat 1) received ciclosporin A in addition to systemic steroids. Four patients received one or more antibiotic treatment lines; of these patients, three showed signs of infection including erysipelas, fever, and a pharyngeal abscess (**Table 2**). Two patients received antimycotic treatment, and one an additional antiviral therapy. The duration of neutropenia was longer in all four patients who received corticosteroids (median 11 days) than in the six patients who did not receive corticosteroids (median 8 days). Because of neutropenia, ICI therapy was interrupted in four patients and permanently discontinued in three patients. Most patients (eight of 10) displayed a normalization of the neutrophil count (**Figure 3**). One patient died due to neutropenia (**Figure 3**, Pat 3). Four patients were re-exposed to ICI after resolution of neutropenia. Two continued with the same PD-1 inhibitor, one patient who had initially received combined PD-1 and CTLA-4 therapy only continued PD-1 monotherapy, and one patient with initial PD-1 monotherapy received anti-CTLA-4 therapy. None of these patients had a relapse of neutropenia, one of the patients had received metamizole before but not at re-exposure (**Table 2**).

### Metamizole-Induced Grade 4 Neutropenia

Patients who received ICI and metamizole (ICI+/met+, n=5) developed neutropenia earlier after starting ICI than patients who did not receive metamizole (ICI+/met-, n=5). Median time to neutropenia onset was 32 days [range 10–51] for ICI+/met+, compared with 48 days [range 11–344] for ICI?A3B2 show $132#?>+/met-. In ICI+/met+ patients, the median time from starting metamizole to onset of neutropenia was 8 days (range 2–43). Patients who received metamizole without ICI (ICI-/met+, n=5) showed a median time to neutropenia onset of 3 days (range 0–1369) after starting metamizole. The duration of grade 4 neutropenia was longer in ICI+/met+ patients (median 11 days, range 4–32 days) than in ICI+/met- patients (median 8 days, range 3–13 days, **Supplemental Table 1**). Non-melanoma and non ICI-treated patients with confirmed metamizole-induced grade 4 neutropenia (ICI-/met+) showed a median duration of neutropenia of 13 days (range 6–28 days; **Supplemental Table 1**). All received further systemic medication at neutropenia onset. All ICI-/met+ patients developed inflammatory symptoms—some cases of which were severe—and were hospitalized for neutropenia. Regarding diagnostics, all ICI-/met+ patients received a laboratory test and a BMB. All ICI-/met+ patients were treated with G-CSF and broad-spectrum antibiotics. None received systemic steroids. Three patients additionally received antimycotic treatment. Neutropenia resolved in all five patients (**Supplemental Table 2**).

### Case Presentation: ICI- or Metamizole-Induced Neutropenia?

*CASE 1* (Pat 7): A 70-year-old female patient was diagnosed with mucosal melanoma of the vulva in November 2012. Local excision with a concurrent sentinel lymph node biopsy of the right groin followed by a complete lymph node dissection of the left groin revealed one lymph node metastasis (stage IIIC according to the American Joint Committee on Cancer [AJCC] 2017). Adjuvant radiotherapy of the genital and groin regions was performed. Nine months later, she presented with recurrent metastatic *BRAF-*wildtype melanoma with nodal and pulmonary involvement. She was treated with nivolumab as part of the CheckMate-067 trial and had stable disease for 17 months. After a second local recurrence in August 2018 with only incomplete resection, follow-up treatment with imiquimod was performed. One month later pulmonary metastases reappeared, and ICI with ipilimumab and nivolumab was initiated. After two cycles of ICI, therapy was paused because of grade 2 hepatitis. Initially, systemic steroids were not commenced, and detailed laboratory examinations were performed. Grade 4 neutropenia was diagnosed, and the patient was hospitalized. Detailed medical history revealed that the patient had taken metamizole two days before the onset of neutropenia due to a headache (**Figure 3**). After consultation with the hematological department, systemic treatment of 30 million international units (IU) G-CSF subcutaneously (SC) once daily was initiated. Additionally, 500 mg ciprofloxacin twice daily and amphotericin B was started. One day after admission, the patient complained of a sore throat and was presented to the ear, nose, and throat department. Computerized tomography (CT) of the neck revealed a pharyngeal abscess. The abscess was drained, and antibiotic therapy was escalated to ampicillin and sulbactam. A gradual improvement of the patient’s physical fitness and normalization of neutrophils was observed after 5 days. Three months later, CT staging showed pulmonary and mediastinal lymph node progression. Because of a lack of therapy options, the patient was re-exposed to nivolumab monotherapy. It was recommended to avoid metamizole. No recurrence of neutropenia was observed. In this case, the patient’s neutropenia could have been triggered by either combined nivolumab–ipilimumab or metamizole, or even by the combination of the three agents.

*CASE 2* (Pat 5): A 71-year-old male patient was diagnosed with a *BRAF* ^V600R^-mutated metastatic melanoma stage IV in November 2018. His pre-existing conditions included chronic pancreatitis and non-immune-related thrombocytopenia and lymphopenia, for which he had not received systemic treatment. Systemic therapy for melanoma with combined BRAF–MEK inhibitors was started. He developed progressive disease within 3 months, and therapy was switched to nivolumab plus ipilimumab. After 6.9 weeks of treatment, the patient developed a temperature of 38.0°C. The laboratory work-up revealed grade 4 neutropenia, and the patient was hospitalized. He received systemic steroids (8 mg dexamethasone intravenously [IV] three times daily for two days, followed by 1 mg/kg methylprednisolone IV for three days, and 1 mg/kg methylprednisolone orally onwards according to scheme), G-CSF SC once daily for four days, as well as antibiotic treatment with IV piperacillin/tazobactam (4.5 g four times daily for one week) and levofloxacin (500 mg once). A BMB was not performed. After 3 days of treatment, the patient’s neutrophils started to increase, and after 4 days of treatment they normalized. The patient was not re-exposed to ICI and received no further systemic therapy. He died of melanoma 2 months later and 7 months after diagnosis of advanced disease. ICI represents a likely trigger of neutropenia in this case.

## Discussion

This international multicenter retrospective analysis reports on one of the largest cohorts of patients with grade 4 neutropenia who were treated with PD-1 inhibitors alone or in combination with ipilimumab. Our findings show that the incidence of grade 4 neutropenia in ICI-treated patients was very low (less than 0.15%), but when it did occur, it was clinically severe and potentially life-threatening. Most patients with grade 4 irNeutropenia presented with clinical symptoms, most often fever and inflammation of the mouth and throat. Half of the patients received metamizole prior to the onset of neutropenia, and this may have contributed or been causal. Therapy with G-CSF was sufficient to achieve neutrophil recovery in 50% of patients; however, one patient (10%) died despite treatment with G-CSF.

Although hematological irAEs are rare, occurring in less than 1% of patients treated with ICI (12, 13), reports of hematological toxicities have increased, possibly because of a more common use of ICI and improved recognition of hematological side effects (21). Neutropenia is one of the most common hematological toxicities, reported in 17% to 26% of patients with hematological irAEs (12–14). Petrelli et al. conducted a meta-analysis that included PD-(L)1-inhibitor-treated patients with several tumor entities. Among 9324 patients from 47 studies, the incidence of grade 3–5 neutropenia was 1.07%, and of febrile neutropenia 0.45% (22). The reported median onset of neutropenia after starting ICI is 10 to 11 weeks, with a median duration (at grade 2 or worse) of 13 to 16.5 days (12, 14, 19). In our study, the onset of high-grade irNeutropenia was earlier (median 6.4 weeks) and the median duration was shorter (9.5 days). A possible explanation for this difference could be the high percentage of patients treated with combined ICI (70%) and the earlier and more frequent (weekly) laboratory testing of these patients. It is well known that irAEs generally occur earlier in patients receiving the combination regimen than in those receiving nivolumab alone (23). In contrast to the results of Delanoy et al., which showed a recurrence of neutropenia in two-thirds of patients after re-exposure to ICI, and a case described by Boegeholz et al. (12, 19), no recurrence of neutropenia occurred upon re-exposure in our study. This is in line with one case reported by Michot et al. (14). These differing results underscore the uncertain nature of the data and the importance of strategically recording and processing rare side effects (14, 24).

Importantly, four patients (40%) with irNeutropenia in our study had a medical history of previously diagnosed hematological diseases. One (10%) of these patients had B-cell chronic lymphocytic leukemia (CLL), which raises the question whether this could be a risk factor for hematological AEs given the frequent observation of autoimmune-related cytopenias in CLL patients (25). Consistent with our data, the study by Delanoy et al. included three patients (9%) with concomitant B-cell CLL (12). This finding underlines the potential increased risk of hematological immunotoxicity in patients with an underlying mature B lymphoid clone (12). Interestingly, a recent retrospective analysis showed that ICI was efficacious in patients with advanced skin cancer (melanoma and Merkel cell carcinoma) and concomitant hematological malignancies, underlining the potential benefit despite hematological comorbidities (26).

In our study, 50% of patients had concomitant treatment with metamizole for pain therapy. Neutropenia is a rare, well-known side effect of metamizole, and concomitant treatment with metamizole presents a diagnostic challenge in ICI-treated patients. In previous studies (16, 17), the median duration of metamizole treatment before onset of acute neutropenia was short, at only 2 days (16). This is in line with our data, which showed a short median time to neutropenia onset after starting metamizole of 3 days in ICI-/met+ patients, compared with 8 days in patients with additional ICI (ICI+/met+). In contrast, onset of neutropenia after starting ICI was later in ICI+/met- patients (48 days) than in ICI+/met+ patients (32 days). Consistent with these data, the Paul Ehrlich Institute in Germany reported 10 cases of pancytopenia or agranulocytosis after initiation of ICI, with onset of neutropenia after 12 to 274 days (six cases within 12 to 28 days, one case after 85 days, and three cases after 240 to 274 days). Of note, three of 10 had concomitant treatment with metamizole (Bulletin zur Arzneimittelsicherheit, August 4, 2016, Paul Ehrlich Institute and Federal Institute of Drugs and Medical Devices). Taken together, ICI-mediated neutropenia seems more likely to occur later after the start of treatment than metamizole-induced neutropenia. Andersohn et al. reported a median time of 10 days between onset of neutropenia and normalization of neutrophil count for metamizole (16). In our cohort, the median duration of grade 4 neutropenia was 13 days for ICI-/met+ patients, compared with 8 days for ICI+/met- and 11 days for ICI+/met+ patients (**Supplemental Table 1**). Because the median duration of neutropenia only slightly differed between patient groups, a final attribution to the causative drug (ICI or metamizole) was not possible. However, the duration of neutropenia might be prolonged by the application of ICI and metamizole simultaneously, compared with ICI alone.

Bone marrow evaluation is an important diagnostic modality for identifying patients with drug-induced grade 4 neutropenia. Garbe reported an absence of granulopoiesis, a neutrophilic maturation arrest or a hypercellularity with increased myeloid precursors and little maturation in the case of peripheral destruction of neutrophils in patients with non-chemotherapy drug-induced agranulocytosis (17). The presence of promyelocytes or myelocytes in the bone marrow generally indicated a recovery within 7 days (17). Bone marrow biopsies from three patients treated with ICI and concomitant metamizole (ICI+/met+) showed a diverse picture, including depletion of granulocytes and slightly impaired maturation of neutrophilic granulopoiesis (**Figure 1**). This is consistent with previous reports (12, 19, 27) that showed variable findings in BMB of patients with ICI-induced neutropenia, ranging from normocellular marrow to blockade in granulocyte maturation or complete absence of myelopoiesis. The timing of the biopsy after onset of neutropenia could be a reason for these differences. Boegeholz et al. showed small infiltrates of CD8+ predominant lymphocytes and slight lymphocytosis of mostly CD8+ T cells in two patients suffering from neutropenia after ICI (19). We could confirm a slightly lower ratio of CD4+ to CD8+ T cells in ICI–met-induced neutropenia than in met-induced neutropenia, which indicates that CD8+ T cell infiltration could play a role in grade 4 ICI–met neutropenia. The pathogenesis of drug-induced grade 4 neutropenia is not completely understood, although toxic or immunoallergic mechanisms are suspected (17). Toxic mechanisms might rely on polymorphisms of genes that encode enzymes that generate or destroy toxic drug metabolites. Immunoallergic mechanisms are thought to be mediated by drug-dependent or drug-induced antibodies that lead to destruction of the granulocytes in peripheral blood or precursor cells in the bone marrow (17). It has been speculated that irAEs are mediated by autoreactive T cells and antibody-mediated processes (28). Similar to other irAEs, generation of autoreactive T and B cells and a decrease in T regulatory phenotype have been proposed as mechanisms for hematologic toxicities (22).

To date, no uniform treatment recommendations exist for irNeutropenia. Because of its high mortality rate, timely diagnostics and treatment is critical for outcome. Standardized approaches are recommended for management of ir-toxicities (29), but these approaches do not cover neutropenia. Treatment of irAE typically includes downregulation of the immune system with systemic steroids, additional systemic immunosuppressive drugs, and symptomatic therapy depending on the grade of toxicity (11, 23, 30–32). Use of systemic steroids and further immunosuppressants constitutes an additional challenge of neutropenia treatment, because these drugs might act counterproductively during bacterial infections and promote sepsis. In previous reports, ICI-induced neutropenia was complicated by severe infection and febrile neutropenia in 55% to 68% of patients (12, 14, 19), and three patients died of a bacterial or fungal infection (12, 14, 19, 27). There is a consensus that broad-spectrum antibiotics should be immediately administered in cases of febrile neutropenia. In addition, G-CSF should be used until neutropenia resolves (12, 14, 19, 27). Conflicting recommendations exist regarding the use of systemic steroids (12, 14, 19, 27). The recommendations from two French studies (12, 14) advise that, in the absence of firm evidence of their efficacy, corticosteroids should not be given systematically, because they could accentuate the risk of infection. Based on data from their meta-analysis, Boegeholz et al. concluded that treatment with corticosteroids in combination with G-CSF does not seem to worsen outcomes regarding infection complications, and thus constitutes an acceptable initial treatment approach (19). Consistent with previous data reporting normalization of neutrophil counts in 67% to 82% of patients (12, 14, 19), 90% of the ICI-treated patients in our study showed resolution of grade 4 neutropenia. Almost all our patients (90%) had been treated with G-CSF, three of them in combination with corticosteroids. The therapeutic benefit of steroids remains unclear and should be critically discussed in the context of associated infections on a case-to-case basis. If additional medication can be excluded as a trigger and irNeutropenia is confirmed, corticosteroid treatment can be considered.

Limitations of our study are its retrospective nature, including the possibility of underreporting of side effects by the treating physician. Nonetheless, because of the clinical relevance of this topic and lack of information and treatment recommendations for this rare side effect, we believe it is important to report real-world outcomes of melanoma patients with grade 4 neutropenia who have been treated with ICI, with or without concomitant drugs that can cause neutropenia. Bias might occur as only patients treated at a maximum care hospital were included and real incidences might be higher as not all cases of grade 4 neutropenia are severe. Although we could confirm a slightly lower ratio of CD4+ to CD8+ T cells in patients with ICI–met-induced neutropenia than in those with met-induced neutropenia, the number of patients in the study is small, and findings depend on the time of biopsy. These data should therefore be interpreted with caution.

In conclusion, our retrospective study shows that grade 4 neutropenia is a potential rare side effect of ICI treatment, which can be life-threatening. The vast majority of patients with ICI-induced grade 4 neutropenia presented with inflammatory symptoms and responded to G-CSF treatment, with a normalization of the neutrophil count in 90% of patients. Most patients with inflammatory symptoms were treated with antibiotics and/or antimycotic and antiviral therapies. Early recognition, initiation of therapy and management of inflammatory complications can prevent a fatal outcome. Corticosteroids can be considered in combination with G(M)-CSF for treatment of irNeutropenia after other causes of neutropenia have been excluded. If infections are suspected or inflammatory symptoms arise, broad-spectrum antibiotics should be administered promptly.

## Data Availability Statement

The original contributions presented in the study are included in the article/**Supplementary Material**. Further inquiries can be directed to the corresponding authors.

## Ethics Statement

The study including human biological samples and related data was approved by the Ethics Committee of Duisburg-Essen University (19-9075-BO).

## Author Contributions

AZ and LZ designed the methodology in collaboration with LH, RK, and MH. AZ and LZ wrote the manuscript. AZ and LZ analyzed and interpreted the data. All authors provided data for **Tables 1**–**3**. All authors reviewed the final manuscript.

## Funding

This work was partly funded by the Deutsche Forschungsgemeinschaft (DFG, German Research Foundation) - SCHA 422/17-1 (KFO 337). The SERIO side effect registry is supported by the Foundation immuno-oncology (Stiftung Immunonkologie), Germany, and the Association for the support of the Cancer Center of the University of Erlangen-Nuremberg (Verein zur Förderung des Tumorzentrums der Universität Erlangen-Nürnberg e.V.), Germany.

## Conflict of Interest

AZ received travel support from Novartis, Sanofi Genzyme, and Bristol-Myers Squibb, outside the submitted work. RK has received speaker fees by NG-Akademie GmbH and Hollister Incorporated, outside the submitted work. MS is an honoraria and/or travel grants from Abbvie, Bristol-Myers Squibb, Merck, Merck Sharp & Dohme, Novartis, Pfizer and Sanofi-Aventis. AG: Speaker´s honoraria from Bristol-Myers Squibb, MSD Sharp & Dohme and Roche; intermittent advisory board relationships with Amgen, Bristol-Myers Squibb, Novartis, MSD Sharp & Dohme, Pierre Fabre Pharmaceuticals, Pfizer, Roche and Sanofi Genzyme; travel and congress fee support from Bristol-Myers Squibb, MSD Sharp & Dohme, Novartis, Pierre Fabre Pharmaceuticals and Roche. Clinical studies: Amgen, Array, Bristol-Myers Squibb, GSK, Novartis, Merck, MSD Sharp & Dohme, Pfizer and Roche. BB received speaker fees for BMS, MSD, Novartis. Travel accomodation: BMS, Pierre Fabre. MS is an honoria for advisory board for Sanofi/Regneron and speaker fees für Novartis Pharma AG. GM has received speaker fees from PharmaMar, outside the submitted work. DJ received advisory board honoraria from BMS, Catalyst, Iovance, Jansen, Merck, Novartis, Oncosec, and Pfizer, and research funding from BMS and Incyte. LS is an honoraria for advisory boards & speaker fees from BMS. CaL received Advisory board: Roche, Novartis, BMS, MSD, SunPharma, Biontech, Kyowa Kirin, Sanofi, Pierre Fabre, Merck; Speakers fee: Roche, Novartis, BMS, MSD, SunPharma, Biontech, Kyowa Kirin, Sanofi, Pierre Fabre, Merck; Travel reimbursement: Roche, Novartis, BMS, MSD, SunPharma, Biontech, Kyowa Kirin, Sanofi, Pierre Fabre, Merck. CP received honoraria (speaker honoraria or honoraria as a consultant) and travel support from: Novartis, BMS, Roche, Merck Serono, MSD, Celgene, AbbVie, Pierre Fabre, SUNPHARMA and LEO. GL is a consultant advisor for Aduro Biotech Inc, Amgen Inc, Array Biopharma inc, Boehringer Ingelheim International GmbH, Bristol-Myers Squibb, Evaxion Biotech A/S, Hexel AG, Highlight Therapeutics S.L., Merck Sharpe & Dohme, Novartis Pharma AG, OncoSec, Pierre Fabre, QBiotics Group Limited, Regeneron Pharmaceuticals Inc, SkylineDX B.V., Specialised Therapeutics Australia Pty Ltd. AM is an honoraria for advisory boards for BMS, MSD, Novartis, Roche, Pierre-Fabre, QBiotics. MC is a consultant advisor for Amgen, BMS, Eisai, Ideaya, MSD, Nektar, Novartis, Oncosec, Pierre-Fabre, Qbiotics, Regeneron, Roche, Merck and Sanofi, and received honoraria from BMS, MSD, and Novartis. CeL received Honoraria: Roche; BMS; Novartis; Amgen; MSD; Pierre Fabre; Pfizer; Incyte; Consulting or Advisory Role: BMS; MSD; Novartis; Amgen; Roche; Merck Serono; Sanofi; Pierre Fabre; Speakers’ Bureau: Roche; BMS; Novartis; Amgen; MSD; Research Funding: Roche; BMS; Travel, Accommodations, Expenses: BMS; MSD; Novartis; Sanofi; Pierre Fabre; Other Relationship: Avantis Medical Systems (board). MT received grants and personal fees from MSD, Ono Pharmaceutical, BMS, Bayer, Eisai, Novartis, Pfizer, Rakuten Medical, Merck Biopharma, personal fees from LOXO, Celgene, Amgen, outside the submitted work. PB received research funding from BeiGene, BMS, MSD and Takeda; advisory board honoraria from Takeda; speakers honoraria from BMS and travel support from Celgene, all outside the submitted work. TEi received speaker fees from Novartis, Pierre Fabre, MSD, Sanofi Genzyme, Alirall Hermal and Bristol-Myers Squibb, outside the submitted work. KK has served as consultant or/and has received honoraria from Amgen, Roche, Bristol Myers Squibb, Merck Sharp and Dohme, Pierre Fabre, and Novartis, and received travel support from Amgen, Merck Sharp and Dohme, Bristol Myers Squibb, Amgen, Pierre Fabre, Medac and Novartis. RG: Invited speaker: Roche, BMS, MSD, Novartis, Amgen, Merck Serono, Almirall Hermal, SUN, Sanofi, Pierre-Fabre. Advisory board: BMS, Roche, Novartis, Almirall Hermal, MSD, Amgen, SUN, Sanofi, Pierre-Fabre, 4SC, Bayer, MerckSerono, Pfizer, Immunocore. Research grants: Novartis, Pfizer, Johnson & Johnson, Amgen, Merck-Serono, SUN Pharma, Sanofi. Travel/meeting support: Roche, BMS, SUN, Merck-Serono, Pierre-Fabre, outside the submitted work. CB received advisory board honoraria and/or speaker´s fees from Amgen, BMS, Immunocore, Merck, MSD, Novartis, Pierre Fabre, Regeneron, Roche, and Sanofi-Aventis. SU declares research support from Bristol Myers Squibb and Merck Serono; speakers and advisory board honoraria from Bristol Myers Squibb, Merck Sharp & Dohme, Merck Serono, Novartis and Roche, and travel support from Bristol Myers Squibb, Merck Sharp & Dohme, and Pierre Fabre. JB is receiving speaker’s bureau honoraria from Amgen, Pfizer, Recordati and Sanofi, is a paid consultant/advisory board member/DSMB member for Almirall, Boehringer Ingelheim, InProTher, ICON, MerckSerono, Pfizer, 4SC, and Sanofi/Regeneron. His group receives research grants from Bristol-Myers Squibb, Merck Serono, HTG, IQVIA, and Alcedis. EL served as consultant and/or has received honoraria from Bristol-Myers Squibb, Merck Sharp & Dohme, Novartis, Medac, Pierre Fabre, Sanofi, Sunpharma and travel support from Amgen, Merck Sharp & Dohme, Bristol-Myers Squibb, Pierre Fabre, Sunpharma and Novartis, outside the submitted work. FM has received travel support or/and speaker’s fees or/and advisor’s honoraria by Novartis, Roche, BMS, MSD and Pierre Fabre and research funding from Novartis and Roche. JH received research support from BMS; advisory board honoraria from Pierre Fabre, Sanofi, Sun Pharma and MSD; speakers honoraria from GSK, Amgen, BMS, MSD, Novartis, Roche, Sanofi and Almirall and travel support from Pierre Fabre and 4SC. DS reports personal fees and non-financial support from Roche/Genentech, grants, personal fees, non-financial support and other from BMS, personal fees from Merck Sharp & Dohme, personal fees and non-financial support from Merck Serono, grant, personal fees and non-financial support from Amgen, personal fees from Immunocore, personal fees from Incyte, personal fees from 4SC, personal fees from Pierre Fabre, personal fees and non-financial support from Sanofi/Regeneron, personal fees from Array BioPharma, personal fees from Pfizer, personal fees from Philogen, personal fees from Regeneron, personal fees from Nektar, personal fees from Sandoz, grants, personal fees and non-financial support from Novartis, personal fees and non-financial support from SunPharma, Replimune, Helsinn, OncoSec and InFlaRx outside the submitted work. LH reports Consultancy, speaker fees, travel grants or research funding: BMS, MSD, Merck, Roche, Amgen, Curevac, Novartis, Sanofi, Pierre Fabre. Clinical studies: BMS, MSD, Merck, Roche, Amgen, GSK, Curevac and Novartis. LZ reports being a consultant and/or receiving honoraria from Roche, Bristol-Myers Squibb, Merck Sharp & Dohme, Novartis, Pierre-Fabre, Sunpharma and Sanofi; research funding to institution from Novartis; and travel support from Merck Sharp & Dohme, Bristol-Myers Squibb, Amgen, Pierre-Fabre, Sanofi, Sunpharma and Novartis, outside the submitted work.

The remaining authors declare that the research was conducted in the absence of any commercial or financial relationships that could be construed as a potential conflict of interest.

## Publisher’s Note

All claims expressed in this article are solely those of the authors and do not necessarily represent those of their affiliated organizations, or those of the publisher, the editors and the reviewers. Any product that may be evaluated in this article, or claim that may be made by its manufacturer, is not guaranteed or endorsed by the publisher.

## Supplementary Material

Supplementary Figure 1 Mann–Whitney *U* test of patients with irNeutropenia (ICI+/met+, n=3) and patients with met-induced neutropenia (ICI-/met+, n=5). ICI, immune checkpoint inhibitors; ir-, immune-related; met, metamizole
