# Supplementary figures and images for: Grade 4 Neutropenia Secondary to Immune Checkpoint Inhibition — A Descriptive Observational Retrospective Multicenter Analysis
Source: Front Oncol. 2021 Oct 21;11:765608. doi: 10.3389/fonc.2021.765608 (PMC8567012; doi:10.3389/fonc.2021.765608)

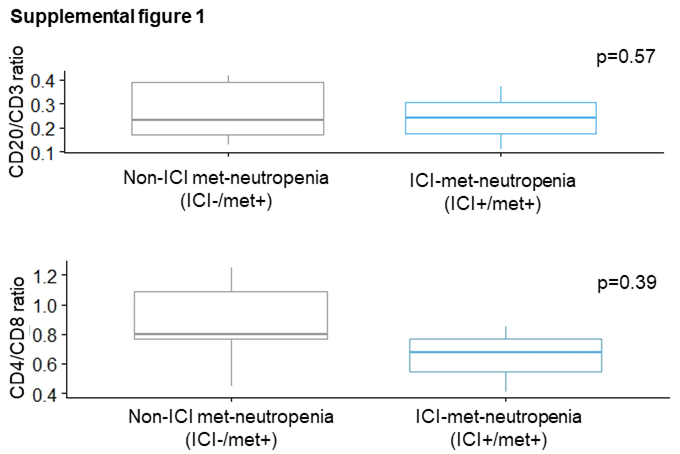

Supplement: Supplementary Figure 1 — Mann–Whitney U test of patients with irNeutropenia (ICI+/met+, n=3) and patients with met-induced neutropenia (ICI-/met+, n=5). ICI, immune checkpoint inhibitors; ir-, immune-related; met, metamizole [file Image_1.tif]
