# Supplementary material for: Grade 4 Neutropenia Secondary to Immune Checkpoint Inhibition — A Descriptive Observational Retrospective Multicenter Analysis
Source: Front Oncol. 2021 Oct 21;11:765608. doi: 10.3389/fonc.2021.765608 (PMC8567012; doi:10.3389/fonc.2021.765608)
Supplement: Supplementary file 2 [file Table_1.docx]

Supplemental Table 1

Characteristics of patients with grade 4 neutropenia

|  | ICI+/met-  (n=5) | ICI+/met+  (n=5) | ICI-/met+  (n=5) |
| --- | --- | --- | --- |
| Median age at onset, years  (range) | 62.2  (53.4–79.8) | 69.9  (28.6–74.1) | 63.6  (21.6–89.2) |
| Median time to neutropenia onset after starting ICI, days  (range) | 48  (11–344) | 32  (10–51) | – |
| Median time to neutropenia onset after starting metamizole, days (range) | – | 8  (2–43) | 3  (0–1369**) |
| Median duration of neutropenia, days  (range) | 8*  (3–13) | 11  (4–32) | 13  (6–28) |

Abbreviations: ICI, immune checkpoint inhibitor; met, metamizole

* One patient died of neutropenia

** Recurrent administration of metamizole documented during this time span; exact time of administration cannot be determined

Supplemental Table 2

Metamizole (met+) treated patients with (ICI+) and without (ICI-) immune checkpoint inhibition-induced grade 4 neutropenia

|  | **ICI+/met+** | | | | | **ICI-/met+** | | | | | |  |
| --- | --- | --- | --- | --- | --- | --- | --- | --- | --- | --- | --- | --- |
|  | **Patient 1** | **Patient 4** | **Patient 7** | **Patient 9** | **Patient 10** | | **Met1** | **Met2** | **Met3** | **Met4** | **Met5** | |
| Age at neutropenia onset, years/sex | 28.4/F | 69.9/M | 70.7/F | 74.1/M | 38.7/F | | 22.6/F | 89.2/F | 36.3/F | 80/F | 63.6/F | |
| Time to neutropenia onset after starting metamizole, days | 43 | 32 | 8 | 6 | 2 | | ~3 | ~2 | ~28 | ~1369 | ~1 | |
| Duration of neutropenia, days | 19 | 32 | 11 | 4 | 7 | | 13 | 28 | 6 | 7 | 24 | |
| Additional drugs | **Metamizole** Denosumab Dimenhydrinate Propranolol Prednisolone | **Metamizole** | **Metamizole** | **Metamizole** | **Metamizole** Lamotrigine Opipramol Ibuprofen Pantoprazole | | **Metamizole**  L- Thyroxin | **Metamizole**  L-Thyroxin  Oxycodone  ASS  Ranexa | **Metamizole**  Amoxicillin  Clindamycin  Ibuprofen | **Metamizole**  Amlodipine  Pantoprazole  Metoprolol  Torasemide  Valsartan  Adenuric  Tiotropium | **Metamizole**  Pantoprazole  Carvedilol  Spironolactone  Thyronajod  Ornithine aspartate  Amitriptyline | |
| Signs and symptoms of neutropenia | Weakness, erysipelas, pain | Mucositis | Pharyngeal abscess | None | Weakness abdominal pain | | Fever  Peritonsillar abscess | Pneumonia  Urinary tract infection | Pneumonia  Capillary leak syndrome  Stomatitis | Pneumonia  (+RSV positive) | Fever  Epigastric pain  Esophageal candidiasis | |
| Diagnosis of neutropenia | Lab BMB | Lab BMB | Lab: differential blood count ENT examination | Lab: differential blood count Autoantibodies BMB Cytogenetic analysis | Lab | | Lab BMB | Lab BMB | Lab BMB | Lab BMB  Respiratory panel | Lab  Bone marrow aspiration BMB  Cytogenetics,  Granulocyte antibodies | |
| Systemic steroids | Prednisolone 1 mg/kg/d (80 mg) IV | None | None | None | None | | None | None | None | None | None | |
| Further treatment | Ciclosporin (175 mg/d) Piperacillin  /tazobactam Vancomycin Ciprofloxacin G-CSF (Filgrastim 30 million. IU/day SC) Valaciclovir (1000 mg/d) Fluconazole (100 mg/d) | G-CSF | G-CSF (Neupogen 30 million IU 1× daily for 6 days)  Ciprofloxacin (500 mg 1-0-1 for 10 days) Unacid 3 g 3× daily (7 days) | G-CSF Ciprofloxacin | G-CSF | | Piperacillin  /tazobactam  Ferritin (anemia)  Konakion  Tranexamic acid  Meronem  G-CSF  Ciprofloxacin | Piperacillin  /tazobactam  Imipenem  G-CSF  Ampho-Moronal  Nitrofurantoin | G-CSF  Piperacillin  /tazobactam  Meropenem  Aciclovir  Fluconazole  Vancomycin  Ceftazidime  Voriconazole  Moxifloxacin  Linezolid | Piperacillin  /tazobactam  Salbutamol/Atrovent inhalation  G-CSF | Piperacillin /tazobactam  G-CSF  Fluconazole | |
| Outcome of neutropenia | Resolved | Resolved | Resolved | Resolved | Resolved | | Resolved | Resolved | Resolved | Resolved | Resolved | |

Abbreviations: ASS, acetylsalicylic acid; BMB, bone marrow biopsy; ENT, ear-nose-throat; G-CSF, granulocyte colony-stimulating factors; ICI, immune checkpoint inhibitor; IFN, interferon α; IU, international units; IV, intravenous; lab, laboratory diagnostics; met, metamizole; RSV, respiratory syncytial virus; SC, subcutaneous
